# Assessment of knowledge, attitudes, and practices of primary healthcare physicians in provinces of Armenia towards hypertension management: a cross-sectional study

**DOI:** 10.1186/s12875-026-03359-6

**Published:** 2026-05-08

**Authors:** Ania Baghoomian, Bowen Zhang, Lorky Libaridian, Marine G. Hovhannisyan, Tatevik Hovakimyan, Shant Shekherdimian

**Affiliations:** 1 Institute for Society and Genetics (ISG), University of California, Los Angeles, Los Angeles, CA USA; 2 Department of Biostatistics, University of California, Los Angeles, Los Angeles, CA USA; 3 Yerevan, Armenia; 4 Department of Hygiene and Ecology, Yerevan State Medical University, Yerevan, Armenia; 5 Department of Surgery, University of California, Los Angeles, Los Angeles, CA USA

**Keywords:** Attitude, Confidence, Hypertension, Knowledge, Practice, Primary care providers, Priority, Armenia

## Abstract

**Introduction:**

Hypertension is the leading cause of death globally, yet little is known about how Armenian primary care providers (PCPs) manage the condition. This study assessed the knowledge, attitudes, practices, priorities, and confidence patterns of PCPs in Armenia towards hypertension management.

**Methods:**

A population-specific proportional sampling was conducted through a cross-sectional survey among PCPs in all provinces of Armenia. A World Hypertension League questionnaire was adapted to follow the Armenian hypertension guidelines. The survey included the following domains: knowledge of Armenian guidelines, attitudes towards management, management practices, priority towards management, and confidence in clinical duties. For each domain, composite scores were calculated as the percentage of items answered correctly or desirably, and regression analyses were performed to examine factors associated with domain-level composite scores and selected individual survey items.

**Results:**

The composite knowledge score of 46.4% (95% CI 45.0%–47.7%%) was suboptimal, despite awareness of hypertension as a priority (97.6%). Composite scores indicated moderate confidence (59.2%, 95% CI 55.1%–63.4%), positive attitude (53.6%, 95% CI 51.4%–55.7%), strong practice (75.2%, 95% CI 74.6%–75.9%), and a high priority (93.1%, 95% CI 92.2%–94.0%). About 45% (*n* = 152) of PCPs adhered to the Armenian hypertension guidelines. Although 92% of PCPs indicated that their clinic has a registry of patients with hypertension, only 30% had confidence in using one. PCPs who participated in the U.S. Agency for International Development (USAID) training program had 1.89 times higher cumulative odds of spending more time with each patient (95% CI: 1.22–2.93, *p* = 0.004). Those who completed an internal medicine (IM) residency had 2.04 times higher cumulative odds of spending more time with each patient compared to those trained in family medicine (FM) (95% CI: 1.26–3.32, *p* = 0.004). PCPs who completed IM residencies had lower practice and priority scores than those in FM. PCPs near the capital had 88% lower odds of referring patients early to specialists compared to those in non-contiguous provinces (OR = 0.12, 95% CI: [0.07, 0.21], *p* < 0.001).

**Conclusions:**

PCPs in Armenia prioritize hypertension and report desirable practices, but gaps in their knowledge, lifestyle counseling, and confidence in registry use remain. These findings underscore the need for additional training, policies to reduce barriers, and further studies to elucidate the reasons behind the “know-do” gap.

**Supplementary Information:**

The online version contains supplementary material available at 10.1186/s12875-026-03359-6.

## Background

Hypertension is the leading global cause of premature death, affecting over 1.28 billion adults [1]. Two-thirds of adults with hypertension live in low- and middle-income countries, but less than one in five achieve adequate control [1]. Hypertension is considered a modifiable metabolic risk factor and thus requires effective management strategies.

The Republic of Armenia is a landlocked middle-income country with a population of 2.9 million people. Armenia consists of eleven provinces, including Yerevan, the urban capital, which accounts for approximately 35% of the population [2, 3]. The remaining ten provinces are predominantly rural, accounting for 65% of the population. Following its independence from the Soviet Union, Armenia’s healthcare system has undergone significant evolution; however, it retains many elements of the Soviet Semashko model, which, among other attributes, relied heavily on specialists, leaving the primary care and public health sectors relatively underdeveloped [4]. Primary health care (PHC) in Armenia is managed through urban polyclinics and rural PHC centers [5].

Armenians have a 1 in 5 chance of premature death due to non-communicable diseases (NCD) [5, 6]. In fact, the World Health Organization’s (WHO) surveillance data have recognized that, despite affecting 63.2% of Armenia’s population aged 45–69, only 6.6% of individuals with hypertension achieve adequate blood pressure control [5]. Globally, non-communicable diseases are managed by frontline primary care providers (PCPs), who interact most frequently with patients. However, although a significant portion of the population receives care through a PCP, hypertension management remains suboptimal in Armenia [2]. The reasons for the inability to achieve blood pressure control are multifactorial and poorly elucidated. Armenia has national hypertension guidelines developed and maintained by the Ministry of Health. These guidelines are generally aligned with international ones, though some discrepancies exist (e.g., higher threshold for diagnosis of hypertension [> 140/90 mmHg], initiation of treatment [> 140/90 mmHg], and systolic blood pressure goals in older patients [130-140mmHg] when compared to international guidelines) [5, 6]. Systemic challenges include inadequate education and training of providers, a lack of provider incentivization, mistrust of the healthcare system, the absence of an effective electronic health records system, and financial barriers, particularly to medications [4, 7].

In 2022, the Ministry of Health in Armenia initiated a primary care strengthening program to revitalize the healthcare sector [2]. Prior to this initiative, primary care had largely remained neglected, except for the U.S. Agency for International Development (USAID) Primary Healthcare Reform Program, which was implemented in 1992 [8, 9]. This program aimed to transform primary care from specialist-driven services in polyclinics into a family medicine (FM) model, retraining over 700 physicians nationwide [8, 9]. Moving forward, understanding the current knowledge base and practice patterns of physicians is essential for developing targeted interventions and implementing sustainable solutions. Therefore, this study aims to understand the knowledge, attitudes, practices, priorities, and confidence of primary care physicians (PCPs) to serve as a foundation for evidence-informed policymaking.

## Methods

### Study population

This cross-sectional survey targeted all 10 provinces of Armenia, where 714 primary care physicians (PCPs), comprising 526 family physicians and 188 local therapists, practice across 314 PHC facilities. A representative sample size of 327 PCPs was calculated using the Cochran formula adjusted for finite populations. This calculation assumed a 95% confidence level, a 4% margin of error, and a 50% expected prevalence rate. To ensure balanced representation of urban and rural physicians, the sample was adjusted to 328 PCPs, stratified proportionally across provinces into 164 urban and 164 rural participants. A comprehensive list of PHC facilities, categorized by province and locality (urban or rural), was obtained from the Health Information Analytical Center of Armenia’s National Institute of Health. Urban facilities, defined as healthcare centers located in cities, ranged from 4 to 13 per province. Urban PCPs were evenly allocated across facilities within each province. In contrast, rural PCPs, typically one per facility (defined as healthcare centers in villages), were selected proportionally from the lists of village facilities. Participants were randomly selected using a random number generator. To ensure accurate survey comprehension, the eligible participants included actively practicing, licensed PCPs in Armenia who were fluent in either Armenian or English.

### Survey design

Based on the confidence-conviction model, the World Health League (WHL) survey design was adapted in accordance with the Armenian Hypertension Guidelines to ensure local relevance and applicability [10]. This survey has been previously validated to determine if additional training is needed to enhance and improve the management of hypertension. The questionnaire was adapted in accordance with the Armenian hypertension guidelines and translated into the Armenian language (Supplemental Digital Content 1, https://perma.cc/K8N3-SEP9). An Armenian medical professional revised the translation to enhance its accuracy and quality. The survey was designed using the Qualtrics platform and administered as an interviewer-led online survey.

The questionnaire included the following sections: (1) demographics of respondents; (2) knowledge and training in Armenian guidelines; (3) attitudes toward hypertension management; (4) current hypertension management practices; (5) priority toward hypertension management; and (6) confidence in performing hypertension management activities. The translated survey was deployed during the pilot study and was revised according to the feedback provided. Additional questions were included to assess consultation times and referral patterns. The providers were instructed to select the single best option that reflected their usual practice. Before administering the survey in July–September 2024, verbal informed consent was obtained and documented by surveyors on the Qualtrics platform. The survey was verbally administered through the Qualtrics platform by designated surveyors. To ensure consistency, the instructions on how to conduct the questionnaire were provided during a meeting, allowing surveyors to clarify any questions they had before deployment.

### Statistical analysis

All questions were multiple-choice and required a single best response unless specified otherwise. Each response option was classified as either “correct,” “incorrect,” “desirable,” or “undesirable.” The proportion of respondents who selected the correct or desirable answer for each question was reported along with the corresponding 95% confidence interval using the Clopper-Pearson method. To summarize responses across domains, a composite score was calculated for each domain by dividing the number of correct or desirable responses by the total number of items in that domain. The resulting scores were expressed as percentages. Bloom’s cutoff points were calculated in this study. In accordance with prior KAP studies, thresholds of “good” (80–100) and “moderate” (60–79) were categorized as adequate, while thresholds of “poor” (< 60) were categorized as limited.

To assess the association between demographic characteristics and scores for each domain, we conducted separate multivariate linear regression analyses for each score. Each model included the following covariates: age group, gender, participation in the USAID training program, province location (contiguous to the capital vs. not), residence (urban vs. rural), primary practice site, specialty of completed residency training, and years of clinical practice.

In addition to domain-level score analyses, we examined two individual survey items not included in the composite scores. The first item, “In one follow-up consultation, how much time do you usually spend on each patient with hypertension?“, had ordinal response options: <5 min, 5–10 min, 10–15 min, 15–20 min, and > 20 min. We analyzed this outcome using a proportional odds model (ordinal logistic regression) to evaluate the association between time spent and physician characteristics.

The second item, “When do you typically refer patients to a cardiologist?“, offered four response options: (1) patient has hypertension with complications (e.g., stroke, myocardial infarction); (2) patient at risk of developing hypertension; (3) patient has a confirmed diagnosis of hypertension; and (4) patient with uncontrolled hypertension on one medication. Option 1 was classified as an appropriate referral, while the remaining options were considered early. The providers were instructed to select the single best option that reflected their usual referral behavior. A binary logistic regression model was used to identify factors associated with early referral practices.

All analyses were conducted using R software (version 4.3.3). Linear regressions were performed using the lm() function, ordinal regressions using the clm() function from the ordinal package, and logistic regressions using the glm() function with a binomial link. Results of (ordinal) logistic regression are presented as log-odds ratios (logORs) with corresponding 95% confidence intervals. For interpretability, odds ratios (ORs) can be obtained by exponentiating the reported coefficients. For both analyses, confidence intervals were constructed at the 95% level, and statistical significance was defined as a p-value less than 0.05.

## Results

A total of 339 primary care providers (PCPs) participated in this study. The majority of providers were female (86.1%, *n* = 292), and most (81.7%, *n* = 277) were 45 years or older. The providers were mainly from urban settings (56.9%, *n* = 193). Most of the providers completed a residency in family medicine (62.8%, *n* = 213), followed by internal medicine (26.3%, *n* = 89). Baseline characteristics of the study participants, including demographics, professional background, and clinical experience, are summarized in Table 1.

**Table 1:** Baseline characteristics of participants by demographics and professional profile

Characteristics	*N*	%
Total	339	100
Gender		
Female	292	86.1
Male	47	13.9
Age group (yr)		
25–34	22	6.5
35–44	40	11.8
45–54	100	29.5
55–64	122	36.0
65+	55	16.2
Residence		
Urban	193	56.9
Rural	146	43.1
Practice site		
Rural	187	55.2
Primary Health Center	98	28.9
Village health posts	89	26.3
Urban	152	44.8
Polyclinics	79	23.3
Medical Center (inpatient/outpatient)	73	21.5
Residency program completed		
Family medicine	213	62.8
Internal medicine	89	26.3
Other	37	10.9
Province		
Aragatsot	26	7.7
Ararat	47	13.9
Armavir	43	12.7
Gegharkunik	26	7.7
Kotayk	65	19.2
Lori	33	9.7
Shirak	45	13.3
Syunik	22	6.5
Tavush	24	7.1
Vayots Dzor	8	2.4
Length of clinical experience (yr)		
< 5	20	5.9
5–10	15	4.4
10–15	28	8.3
15–20	47	13.9
> 20	229	67.6
Participated in the USAID retraining program		
Yes	231	68.1
No	108	31.9
Frequency of managing patients with hypertension		
Less than once a month	6	1.8
Once a month	18	5.3
A few times a month	16	4.7
About once a week	18	5.3
A few times a week	37	10.9
Every day	244	72.0
Hypertension guidelines used		
Armenian hypertension guidelines	152	44.8
European hypertension guidelines	153	45.1
Other guidelines	23	6.8
Do not follow any guidelines	11	3.2
Time usually spent on each patient with hypertension in one follow-up examination (min)		
< 5	6	1.8
5–10	68	20.1
10–15	157	46.3
15–20	77	22.7
> 20	31	9.1
Typical referral reason for cardiology		
Patient has hypertension with complications (stroke, heart attack, etc.)	225	66.4
Patient who has uncontrolled hypertension with one medication	48	14.2
Patient at risk of developing hypertension	44	13.0
Patient has a confirmed diagnosis of hypertension	22	6.5

### Knowledge

Knowledge-related responses are summarized in Table 2. Nearly all providers (97.6%, *n* = 331) correctly identified hypertension as a priority problem in their catchment population. While 76.1% (*n* = 258) reported following the Armenian Hypertension Guidelines, only 45% (*n* = 152) identified these guidelines as their primary reference. Of those who reported having a physical copy of the guidelines (64.3%, *n* = 218), most (59.6%, *n* = 202) indicated that they could display how to access them. Knowledge of hypertension epidemiology in Armenia was suboptimal. Notably, only a small portion of providers were aware of epidemiological data on hypertension in Armenia. Regarding the diagnosis of hypertension, 56% (*n* = 190) and 54% (*n* = 183) of providers correctly identified the lowest levels of systolic and diastolic blood pressure, respectively, as hypertensive. While 80% (*n* = 287) of providers correctly identified that most patients require two or more antihypertensive medications, knowledge about lifestyle recommendations for lowering blood pressure was relatively low (Table 2). Only 36.9% (*n* = 125) and 1.5% (*n* = 5) correctly identified the recommended daily salt consumption and physical activity recommendations, respectively. The average composite knowledge score among providers was 46.4%, despite considerable awareness of hypertension as a priority (*n* = 331, 97.6%).

**Table 2:** Correct knowledge score about hypertension of primary healthcare physicians (*N* = 339)

Correct knowledge	*N*	%	95% CI
National Hypertension Guidelines			
Correctly identified (Armenian National Hypertension Management guidelines) as the guideline of choice	152	44.8	(39.5–50.3)
Correctly identified hypertension as a priority problem in the catchment population	331	97.6	(95.4–99.0)
Follows the Armenian Hypertension Guidelines in treating hypertensive patients	258	76.1	(71.2–80.5)
Has a copy of the guidelines	218	64.3	(59.0–69.4)
Able to show where to access the guidelines	202	59.6	(54.2–64.9)
Epidemiology of hypertension in Armenia			
Correctly identified overall prevalence percentage of hypertension among adults aged 30–79 is 47%	72	21.2	(17.0–26.0)
Correctly identified percentage of adults aged 30–79 diagnosed with hypertension is 41%	81	23.9	(19.5–28.8)
Correctly identified among hypertensive patients, the percentage of people who are treated is 28%	73	21.5	(17.3–26.3)
Correctly identifying those with controlled hypertension is 9%	24	7.1	(4.6–10.4)
Diagnosis of hypertension			
Correctly identified the lowest systolic blood pressure level to be ‘hypertensive’ as 140 mmHg	190	56.0	(50.6–61.4)
Correctly identified the lowest level of diastolic blood pressure to be ‘hypertensive’ as 90 mmHg	183	54.0	(48.5–59.4)
Management of hypertension with regard to lifestyle recommendations and drug therapy			
Correctly identified that most people with hypertension require two or more antihypertensive drugs to achieve blood pressure control	287	84.7	(80.4–88.3)
Correctly identified less than 5 g as the recommended daily level of salt consumption for adults with hypertension	125	36.9	(31.7–42.3)
Correctly identified, it is recommended that people with hypertension obtain at least 150 min of moderate physical activity per week, accumulated across most days	5	1.5	(0.5–3.4)
Composite Score*	339	46.4	(45.0–47.7)

*Composite score was calculated as the proportion of correct or desirable responses out of the 14 items (all items had a defined correct or desirable response)

### Attitude

Table 3 presents providers’ attitudes toward hypertension management. Most providers believed that patients’ difficulty in accessing clinics and providers’ de-prioritization of hypertension relative to other tasks had little to no impact on hypertension management, with 61% (*n* = 207) and 51% (*n* = 174) reporting no effect, respectively. Most providers disagreed that patients’ perception of importance, unwillingness to follow lifestyle changes, and inability to afford medications had little to no impact on hypertension management (13.9%, *n* = 47), (17.1%, *n* = 58), and (26.3%, *n* = 89), respectively. Only 31.6% (*n* = 107) believed that the lack of consultation has little to no impact on hypertension management.

**Table 3:** Desirable attitude score about hypertension of primary healthcare physicians (*N* = 339)

Desirable attitude (correctly identified/desirably considered)	*N*	%	95% CI
Desirably considers the following barrier to have little or no impact on hypertension management:			
Lack of time for consultation	107	31.6	(26.6–36.8)
Hypertension is not significant enough compared to other work that needs to be done	174	51.3	(45.9–56.8)
The patients do not think it is important	47	13.9	(10.4–18.0)
Patients are not willing to follow lifestyle changes	58	17.1	(13.3–21.5)
Patients cannot afford drug treatment for hypertension	89	26.3	(21.6–31.3)
Patients have difficulty accessing the clinic	207	61.1	(55.6–66.3)
Hypertension control			
Correctly identifies that it is not better to use no drugs or as few drugs as possible, even if that means hypertension is not controlled	239	70.5	(65.3–75.3)
Correctly identifies the patient should agree with the blood pressure target and treatment plan	285	84.1	(79.7–87.8)
Correctly identifies that drug therapy should not only be used if the patient is willing to change their lifestyle	193	56.9	(51.5–62.3)
Correctly identifies that drug therapy should not only be used if the patient is not willing to change their lifestyle	189	55.8	(50.3–61.1)
Correctly identifies counseling about lifestyle interventions to prevent and control hypertension should be advised to all patients	308	90.9	(87.3–93.7)
Correctly identifies all the health care professionals assisting the patient should try to achieve the same target blood pressure	202	59.6	(54.2–64.9)
Correctly identifies if the blood pressure target is not achieved, more drug treatment should be sequentially added	262	77.3	(72.5–81.6)
Composite Score*	339	35.6	(51.4–55.7)

*Composite score was calculated as the proportion of correct or desirable responses out of the 13 items (all items had a defined correct or desirable response)

A high percentage (90.9%, *n* = 308) correctly identified that lifestyle intervention counseling should be advised for all patients. Additionally, 84.1% (*n* = 285) recognized that patients should agree with their blood pressure targets and treatment plans. Most providers (70.5%, *n* = 239) correctly identified that minimizing medication use at the expense of blood pressure control was not appropriate. Regarding treatment strategies, 77.3% (*n* = 262) correctly identified that the sequential addition of drugs was appropriate when blood pressure targets were not achieved. There was moderate agreement (59.6%, *n* = 202) that all healthcare professionals should work toward the same blood pressure targets.

### Practice

Regarding practice patterns shown in Table 4, nearly all providers reported “always” or “usually” performing the desired actions, with the exception of recommending the consumption of 5 or more portions of fresh or frozen fruit/vegetables per day (76.4%). In terms of hypertension management, there was considerable variability, with 99% of providers reporting that they advise patients to manage hypertension-related issues in the interim between scheduled visits, while only 14% (*n* = 47) reported using a validated electronic device to measure blood pressure in their practice. Notably, only 57% of providers did not have preferred brand names when prescribing medications. There was similar variation in reported follow-up visit practices, with nearly all providers (99%) referring an undiagnosed asymptomatic patient with a blood pressure of 224/112 to the hospital, while scheduling of routine follow-up visits for patients with uncontrolled (12%), controlled (9.4%), and after recent treatment change (13.6%) were much lower.

**Table 4:** Desirable practice score about hypertension of primary healthcare physicians (*N* = 339)

Desirable practice	*N*	%	95% CI
Hypertension management			
Clinic uses a validated electronic device	47	13.9	(10.4–18.0)
Clinic assesses cardiovascular risk	289	85.3	(81.0–88.9)
Clinic has a registry of people with hypertension	312	92.0	(88.6–94.7)
Provider does not have preferred brand names when prescribing medications	193	56.9	(51.5–62.3)
Advises patients to deal with hypertension-related issues in the interim between their scheduled visits	336	99.1	(97.4–99.8)
Actions Always or Usually Performed			
Measure blood pressure to screen for hypertension at all adult visits	335	98.8	(97.0–99.7)
Counsel about hypertension, its adverse effects, and the need for treatment in people with hypertension	337	99.4	(97.9–99.9)
Counsel how to properly measure blood pressure at home in people with hypertension	334	98.5	(96.6–99.5)
Assess cardiovascular risk in people with hypertension	332	97.9	(95.8–99.2)
Counsel about lifestyle interventions to prevent and control hypertension	338	99.7	(98.4–100.0)
Prescribe antihypertensive drugs in people with hypertension	335	98.8	(97.0–99.7)
Prescribe antihypertensive drugs based on cardiovascular risk for patients with hypertension	330	97.3	(95.0–98.8)
Assess adherence to antihypertensive drug therapy at all visits in people with hypertension	336	99.1	(97.4–99.8)
Recommends eating 5 or more portions of fresh or frozen fruit or vegetables a day	259	76.4	(71.5–80.8)
Recommends getting regular physical activity	337	99.4	(97.9–99.9)
Recommends reducing alcohol consumption in heavy Consumers	337	99.4	(97.9–99.9)
Recommends reducing the amount of salt in the diet	338	99.7	(98.4–100.0)
Recommends having a healthy body weight	339	100.0	(98.9–100.0)
Blood pressure follow-up visits			
Scheduling a follow-up visit after 3 months for 44 44-year-old. old asymptomatic patient with a blood pressure of 136/76 mmHg	41	12.1	(8.8–16.0)
Scheduling a follow-up visit after 1–2 weeks for 44 44-year-old. old undiagnosed, asymptomatic patient with a blood pressure of 152/96 mmHg	171	50.4	(45.0–55.9)
Scheduling a follow-up visit after 1–2 weeks for 44 44-year-old. old undiagnosed, asymptomatic patient with a blood pressure of 168/108 mmHg	199	58.7	(53.3–64.0)
Immediate referral to hospital for a 44-year-old. old undiagnosed asymptomatic patient with a blood pressure of 224/112 mmHg	268	79.1	(74.3–83.3)
Immediate referral to hospital for a 44-year-old. old undiagnosed patient with headaches, blurred vision, and a blood pressure of 224/112 mmHg	335	98.8	(97.0–99.7)
Scheduling a follow-up visit after 2–4 weeks for a new patient with a recent diagnosis of hypertension, just starting treatment	76	22.4	(18.1–27.2)
Scheduling a patient for a routine visit after three months when blood pressure is controlled	32	9.4	(6.5–13.1)
Scheduling a patient for a follow-up visit 2–4 weeks after a treatment change for uncontrolled blood pressure control	46	13.6	(10.1–17.7)
Composite Score*	339	75.2	(74.6–75.9)

*Composite score was calculated as the proportion of correct or desirable responses out of the 26 items (all items had a defined correct or desirable response)

### Priority

Priority-related responses are summarized in Table 5. In general, desirable priority scores were high. With the exception of utilizing validated devices (78.8%, *n* = 267) and measuring blood pressure in both standing and sitting positions (75.8%, *n* = 257), providers reported the remaining practices as “most important” more than 88% of the time. Similarly, there was near-universal prioritization of counseling about hypertension’s adverse effects, lifestyle change, and drug treatment, except for the practice of providing written information to patients (*n* = 269, 79.4%). Notably, 85.5% (*n* = 290) prioritized having a registry with regular reports of hypertension diagnosis, treatment, and control, while 93.5% (*n* = 315) prioritized using a protocol to manage hypertension.

**Table 5:** Desirable priority scores about hypertension of primary healthcare physicians (*N* = 339)

Priority practice (desirably considered as most important)	*N*	%	95% CI
Accurate blood pressure measurement			
The person conducting the reading has been trained and can accurately assess blood pressure tests within the last year	300	88.5	(84.6–91.7)
A validated electronic blood pressure device is used	267	78.8	(74.0–83.0)
The patient is rested for 5 min in a quiet, comfortable place before the measurement	335	98.8	(97.0–99.7)
An upper arm cuff that is the correct size for the patient’s arm is used	335	98.8	(97.0–99.7)
The patient’s arm is supported at the heart level	326	96.2	(93.5–97.9)
Blood pressure readings are taken in the sitting and standing positions	257	75.8	(70.9–80.3)
The blood pressure is assessed at the initial visit in both arms, and the arm with the higher blood pressure is used subsequently	324	95.6	(92.8–97.5)
Counseling about the adverse effects of hypertension, lifestyle change, and drug treatment			
Involving family members in the counseling	312	92.0	(88.6–94.7)
Providing written, in addition to verbal, information	269	79.4	(74.6–83.5)
Encouraging the patient to ask questions	324	95.6	(92.8–97.5)
Providing information about the prognosis and the need for treatment at a level the patient understands	335	98.8	(97.0–99.7)
That the benefits of the treatment and the low probability of adverse effects are understood by the patient	336	99.1	(97.4–99.8)
That serious and more common adverse effects are explained to the patient, and the patient knows how to report them	334	98.5	(96.6–99.5)
That the importance of adherence to lifelong therapy, even when hypertension is controlled, is understood by the patient	335	98.8	(97.0–99.7)
That close follow-up is scheduled until blood pressure is controlled, and then regular follow-up visits are scheduled	335	98.8	(97.0–99.7)
That the patient can afford and implement the therapeutic plan	331	97.6	(95.4–99.0)
Potential barriers to lifestyle and drug adherence are asked about, and solutions are developed with the patient	335	98.8	(97.0–99.7)
Protocols and registries for hypertension management			
Prioritizes using a protocol to manage hypertension	317	93.5	(90.3–95.9)
Prioritizes having a registry with regular reports on hypertension diagnosis, treatment, and control	290	85.5	(81.3–89.1)
Composite Score*	339	93.1	(92.2–94.0)

*Composite score was calculated as the proportion of correct or desirable responses out of the 19 items (all items had a defined correct or desirable response)

### Confidence

Table 6 summarizes providers’ confidence in hypertension management activities. Confidence scores were generally lower and more variable, ranging from 70.2% (*n* = 238) for extreme or high confidence in accurately measuring blood pressure to 30.4% (*n* = 103) for extreme or high confidence in implementing registry utilization. Confidence in the utilization of treatment algorithms and protocols was also low (33.9%, *n* = 105).

**Table 6:** Desirable confidence scores about hypertension of primary healthcare physicians (*N* = 339)

Practice with extreme/high confidence	*N*	%	95% CI
Measuring blood pressure accurately for all adult patient visits to screen for hypertension	238	70.2	(65.0–75.0)
Accurately diagnosing hypertension	217	64.0	(58.7–69.1)
Counseling about hypertension and its adverse effects, and the need for treatment	228	67.3	(62.0–72.2)
Counseling about lifestyle interventions to prevent and control hypertension	235	69.3	(64.1–74.2)
Prescribing antihypertensive drugs	215	63.4	(58.0–68.6)
Achieving target blood pressures with lifestyle and or drug treatment	215	63.4	(58.0–68.6)
Counseling about antihypertensive drug therapy and adherence to drugs	224	66.1	(60.8–71.1)
Assessing adherence to antihypertensive drug therapy at each visit in people prescribed antihypertensive drugs	217	64.0	(58.7–69.1)
Implementing and using a registry that has regular reports on hypertension diagnosis, treatment, and control	103	30.4	(25.5–35.6)
Implementing and using a treatment algorithm or pathway in the clinic	105	33.9	(28.9–39.2)
Composite Score*	339	59.2	(55.1–63.4)

*Composite score was calculated as the proportion of correct or desirable responses out of the 10 items (all items had a defined correct or desirable response)

The highest confidence was observed in the provider’s ability to accurately measure blood pressure for screening hypertension (70.2%, *n* = 238). Similarly, providers had high confidence (66.1%, *n* = 224) in counseling and prescribing antihypertensive drug therapy to their patients. Furthermore, 64.0% (*n* = 217) reported confidence in accurately diagnosing hypertension and assessing medication adherence during follow-up visits. In addition, 63.4% (*n* = 215) expressed confidence in achieving target blood pressures through lifestyle, drug interventions, and prescribing antihypertensive drugs. Only 33.9% (*n* = 115) expressed confidence in implementing treatment algorithms or pathways in their clinics. Approximately 30.4% (*n* = 103) felt confident in implementing and using a registry system to track hypertension diagnosis, treatment, and control.

### Associations between provider characteristics and KAP domains

The direction and strength of associations between provider characteristics and KAP-related domains are summarized in Table 7. Across domains, several provider characteristics were consistently associated with differences in performance.

**Table 7:** Direction and strength of associations between provider characteristics and KAP-related domains in hypertension management, based on linear regression models

Domain	Practice location	USAID participation	Practice site	Residency program (Ref. family medicine)	
	Province adjacent to the capital	Yes	Urban	Internal medicine	Other program
Knowledge	-		-	-	-
Attitude		-		--	--
Practice				-	
Priority				-	
Confidence	++	--		--	--

This table summarizes the direction and statistical significance of associations from five separate linear regression models examining Knowledge, Attitude, Practice, Priority, and Confidence related to hypertension management. Plus (+) and minus (–) signs indicate statistically significant positive and negative associations, respectively (*p* < 0.05). Double signs ( + + or --) indicate stronger statistically significant associations (e.g., absolute coefficient values larger than 10)

Geographic location was associated with divergent patterns across domains. Physicians practicing in provinces contiguous to the capital had significantly lower knowledge scores but higher confidence compared to those in non-contiguous provinces.

Training background also showed consistent associations. Compared to providers trained in family medicine, those who completed internal medicine or other residency programs had lower scores across multiple domains, especially in knowledge, attitude, and confidence.

Participation in the USAID retraining program was negatively associated with both attitude and confidence scores, suggesting a potential gap between training exposure and sustained perceptions or confidence in hypertension management.

Practice setting was additionally associated with knowledge outcomes, with providers working in polyclinics demonstrating lower knowledge scores compared to those practicing in primary health centers.

Overall, these findings indicate that both structural factors (e.g., geographic location and practice setting) and training-related factors (e.g., residency background and retraining exposure) contribute to variability in KAP outcomes among providers.

### Consultation time and referral patterns

**Fig. 1:**
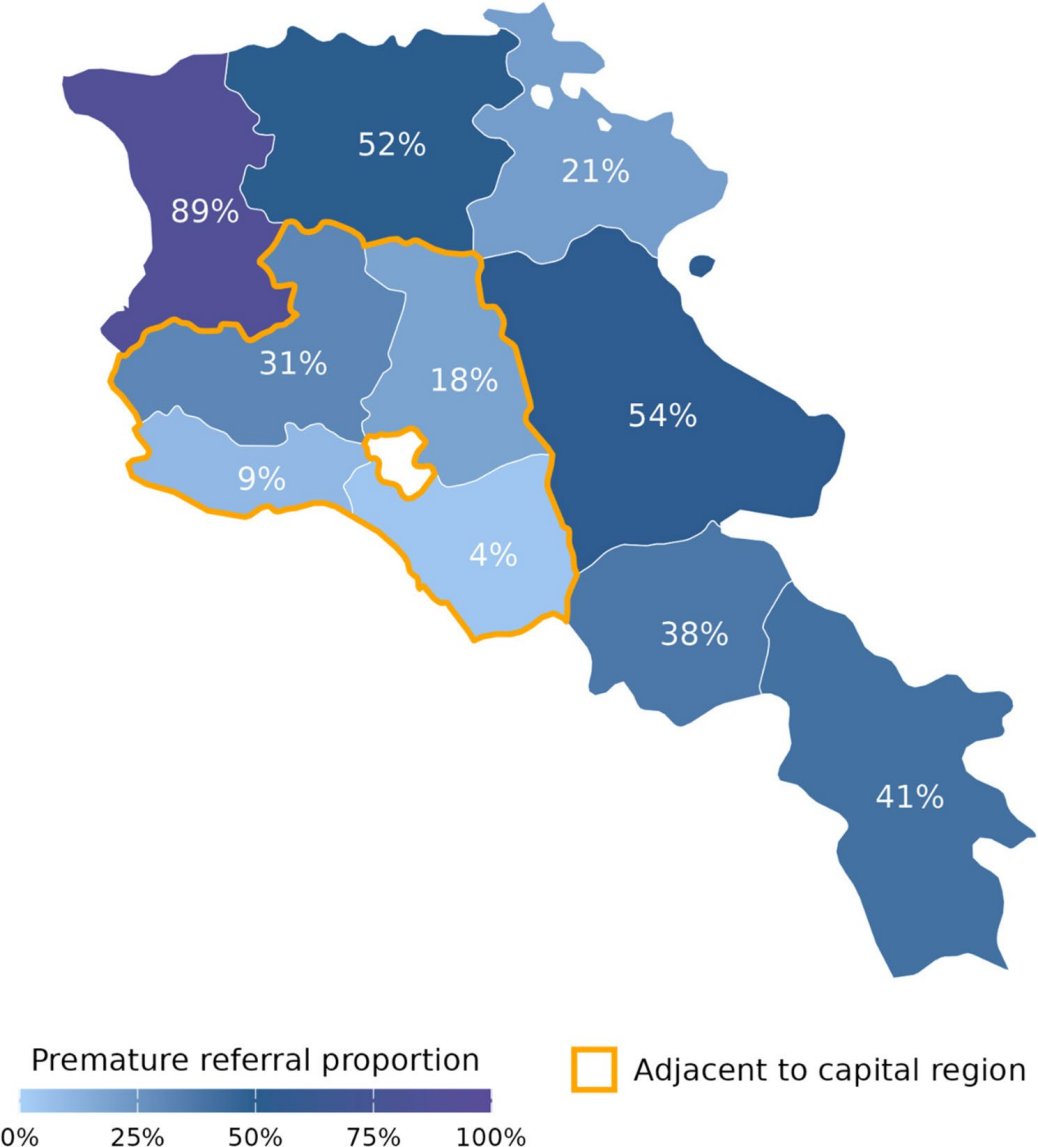
Prevalence of early referral practices across Armenian provinces. The map shows the proportion of surveyed primary care providers in each province who selected an early referral option in response to the question, “When do you typically refer patients to a cardiologist?” Early referral was defined as selecting any response other than “patient has hypertension with complications (e.g., stroke, myocardial infarction).” Provinces outlined in orange are adjacent to the capital region and were included as a covariate in regression analyses

In multivariate analysis, only the type of training and participation in USAID had a significant impact on consultation time (Table 8). Participation in the USAID training program was significantly associated with increased time spent per patient (OR = 1.89, 95% CI: [1.22, 2.93], *p* = 0.004). Completing a residency in IM, as opposed to FM, was significantly associated with increased time spent per patient (OR = 2.04, 95% CI: [1.26, 3.32], *p* = 0.004)(OR = 2.04, 95% CI: [1.26, 3.32], *p* = 0.004) (Table 9). Physicians in provinces contiguous to the capital tended to have fewer early referrals (Fig. 1). Providers in contiguous provinces had 88% lower odds of referring patients compared to those in non-contiguous provinces (OR = 0.12, 95% CI: [0.07, 0.21], *p* < 0.001).

**Table 8:** Association between provider characteristics and time spent on each patient with hypertension from an ordinal logistic regression model

Variable	Log(OR)	95% CI	*p*-value
Age (Ref. 25–34 years)			
35–44 years	0.38	(-0.58–1.35)	0.440
45–54 years	0.33	(-0.59–1.26)	0.486
55–64 years	0.08	(-0.96–1.13)	0.884
65 + years	0.64	(-0.46–1.75)	0.253
Participation in the USAID retraining program (Ref. No.)			
Yes	0.64	(0.20–1.08)	0.004
Practice location (Ref. province not adjacent to the capital)			
Province adjacent to the capital	0.12	(-0.30–0.55)	0.568
Residence (Ref. Urban)			
Rural	0.11	(-0.48–0.70)	0.719
Practice site (Ref. primary care center)			
Medical Center (inpatient/outpatient)	0.08	(-0.63–0.78)	0.826
Polyclinics	-0.02	(-0.69–0.65)	0.951
Village health posts	0.33	(-0.22–0.89)	0.241
Gender (Ref. Female)			
Male	0.46	(-0.14–1.06)	0.133
Length of clinical experience (Ref. < 20 yrs)			
> 20 yrs	-0.44	(-1.09–0.21)	0.188
Residency program completed (Ref. family medicine)			
Internal medicine	0.72	(0.23–1.20)	0.004
Other	0.22	(-0.51–0.94)	0.557

**Table 9:** Association between provider characteristics and early referral practices for hypertension from a logistic regression model

Variable	Log(OR)	95% CI	*p*-value
Age (Ref. 25–34 years)			
35–44 years	0.17	(-1.10–1.48)	0.793
45–54 years	-0.47	(-1.76–0.85)	0.482
55–64 years	-0.42	(-1.86–1.03)	0.564
65 + years	-1.22	(-2.78–0.33)	0.121
Participation in the USAID retraining program (Ref. No.)			
Yes	-0.005	(-0.57–0.57)	0.987
Practice location (Ref. province not adjacent to the capital)			
Province adjacent to the capital	-2.14	(-2.74–-1.57)	< 0.001
Residence (Ref. Urban)			
Rural	-0.36	(-1.10–0.38)	0.332
Practice site (Ref. primary care center)			
Medical Center (inpatient/outpatient)	0.62	(-0.26–1.52)	0.170
Polyclinics	0.38	(-0.44–1.21)	0.366
Village health posts	-0.26	(-1.05–0.52)	0.514
Gender (Ref. Female)			
Male	-0.67	(-1.51–0.11)	0.102
Length of clinical experience (Ref. < 20 yrs)			
> 20 yrs	0.48	(-0.44–1.45)	0.315
Residency program completed (Ref. family medicine)			
Internal medicine	0.46	(-0.15–1.08)	0.141
Other	0.26	(-0.68–1.16)	0.580

## Discussion

This study examined the knowledge, attitudes, practices, and confidence of PCPs in Armenian provinces regarding the management of hypertension. Composite scores indicated low knowledge, moderate confidence, positive attitude, strong practice, and high prioritization. Less than half of PCPs adhered to the Armenian hypertension guidelines. While most providers indicated that their clinic has a registry of patients with hypertension, few had confidence in using one. Providers who participated in USAID training or completed an internal medicine residency spent more time with each patient. Providers in provinces further from the capital tended to refer patients to specialists earlier.

Significant gaps were identified in various domains. Knowledge of lifestyle modification for hypertension control was the most limited. Less than half of the providers correctly identified salt intake recommendations. Moreover, approximately 1.5% of providers could determine the correct physical activity guidelines. Despite physicians’ prioritization of lifestyle counseling, providers lack specific knowledge according to current guideline recommendations. This discrepancy suggests that providers may be counseling patients with incorrect or outdated recommendations, but further studies are necessary to better characterize the quality of provider guidance. Regarding practice patterns, only 14% of providers reported using a validated electronic device to measure blood pressure. Self-reported confidence in the use of clinical adjuncts, such as treatment algorithms and registries, was low. Regarding attitudes, fewer than a third believed that a lack of consultation has little to no impact on the management of hypertension. Similar findings have been reported in KAP studies in Mongolia and Qatar, where providers had gaps in hypertension-related knowledge despite positive attitudes towards hypertension management [11, 12]. In addition, comparable gaps in knowledge and practices have been identified in Namibia. Specifically, over 90% of providers in Namibia identified hypertension as a serious health problem in their population. However, only 39% of the health sciences faculty regularly monitored the blood pressure of patients. Despite the awareness of hypertension as a serious concern, providers demonstrated variable and suboptimal practice and knowledge patterns.

Significant disparities are noted when comparing the results from this survey to prior assessments of actual hypertension diagnoses and treatments in Armenia. Although over 50% of providers correctly identified the lowest levels of systolic and diastolic blood pressure as hypertensive, and nearly all providers reported measuring blood pressure to screen for hypertension at all adult visits, recent findings from the World Bank data indicate a treatment initiation rate of less than 35% [10]. Furthermore, while 85% of providers recognized that most patients with hypertension require more than one medication, and over 90% agreed that patients should be advised on lifestyle modification, a 2016 WHO STEPS survey in Armenia found that only 18% of diagnosed individuals with hypertension had controlled blood pressure [7]. This disparity between clinical knowledge and implemented care is a well-known phenomenon referred to as the ‘know-do gap’ [13, 14]. The physicians’ perception of patient-related barriers may explain this gap. Providers may attribute a patient’s uncontrolled hypertension to personal factors such as a patient’s socioeconomic constraints or limited adherence to medications and effective lifestyle alterations. As a result, these various perceptions may inadvertently lead the providers to deprioritize various clinical responsibilities that they perceive as out of their control. Therefore, providers may demonstrate disparities between their clinical knowledge and the care that they provide to their patients. Further assessments are required to determine the specific reasons for these disparities and to address them effectively.

Additionally, the limitations to effectively utilizing available resources impact the care that providers implement for their patients. The use of registries to facilitate hypertension management and follow-up can help improve patient outcomes, but comfort with using such registries is crucial [15]. Despite 92% of providers indicating that the clinic they work at has a registry of patients with hypertension, only 30% have confidence in implementing and using a registry with regular reports on hypertension diagnosis, treatment, and control. These findings underscore the need to design and tailor competency-based training programs that reflect the current needs of providers.

Providers practicing in provinces contiguous to the capital had significantly lower knowledge scores compared to those in non-contiguous provinces. A possible explanation is that providers closer to the capital are more likely to work in settings where specialists are readily available and may serve as the first point of contact for patients. As a result, regular exposure and interactions with chronic disease management, such as hypertension, may be reduced.

Providers who completed their residency in specialties other than FM had lower knowledge and attitude scores in hypertension management compared to those trained in FM, suggesting differences in training as it pertains to chronic disease management. Further assessments of residency curriculum and training standards may help improve knowledge and attitude scores amongst various primary care residency programs in Armenia.

Providers participating in USAID training had lower attitude and confidence scores than those who did not participate in the training. Most notably, our findings identified that only 30% of providers were confident in using a registry system, and just 33.9% had confidence in implementing a treatment algorithm. These gaps suggest that training initiatives, such as those supported by USAID, may not translate into long-term, sustainable clinical outcomes on their own. While the reasons for this are difficult to elucidate, these findings underscore the importance of robust monitoring and evaluation of all interventions to ensure they achieve and maintain their intended outcomes.

To our knowledge, this is the first study to investigate the KAP of healthcare providers in Armenian provinces regarding the management of hypertension. The study assessed multiple domains, including KAP, which underscored a direct overview of current patterns of care that patients experience in their respective provinces. Furthermore, the use of a standardized questionnaire adapted to the Armenian population provides pertinent insight for future policy development. This study has several limitations. Most notably, the study was based on a self-reported questionnaire; therefore, the providers may have answered the questions in a socially desirable manner. The social desirability bias can alter the providers’ responses to make them more desirable. In addition, while preparatory trainings were held, data collection via survey administrators may have introduced bias [16]. While a previously validated survey tool was utilized, translated by native Armenian speakers, and back-translated to ensure reliability, the survey did not undergo a formal validation in the Armenian language, possibly introducing various forms of bias. For instance, measurement or instrument bias may have occurred if the providers failed to accurately report their clinical behaviors during the questionnaires. Construct validity bias was also possible if the translated surveys did not accurately reflect the concepts intended to be assessed.

## Conclusion

The study highlighted several knowledge gaps among primary care physicians while demonstrating that providers prioritized hypertension with a positive attitude, confidence, and effective practices. The results underscore the need for additional, consistent, high-quality educational and training programs, along with corresponding policies and financing mechanisms that encourage proper implementation of learned knowledge. We recommend robust monitoring and evaluation mechanisms to assess changes to KAP during ongoing reforms.

## Supplementary Information

Supplementary Material 1.

Supplementary Material 2.

## Data Availability

The datasets used and/or analysed during the current study are available from the corresponding author on reasonable request.

## Abbreviations

KAP: Knowledge, attitudes, practices
PCP: Primary Care Provider
PHC: Primary Health Care
FM: Family Medicine
IM: Internal Medicine
USAID: U.S. Agency for International Development
CI: Confidence interval
