# Supplementary material for: Assessment of knowledge, attitudes, and practices of primary healthcare physicians in provinces of Armenia towards hypertension management: a cross-sectional study
Source: BMC Prim Care. 2026 May 8;27:230. doi: 10.1186/s12875-026-03359-6 (PMC13262312; doi:10.1186/s12875-026-03359-6)
Supplement: Supplementary file 1 — Supplementary Material 1. [file 12875_2026_3359_MOESM1_ESM.docx]

**Supplementary Table 1: Association between provider characteristics and knowledge score from a linear regression**

| Variable | Coef. | S.E. | p-value |
| --- | --- | --- | --- |
| Intercept | 44.57 | 3.02 | <0.001 |
| Age (Ref. 25-34 years) |  |  |  |
| 35-44 years | 1.66 | 3.22 | 0.607 |
| 45-54 years | 5.50 | 3.12 | 0.079 |
| 55-64 years | 5.53 | 3.51 | 0.116 |
| 65+ years | 7.27 | 3.72 | 0.052 |
| Participation in the USAID retraining program (Ref. No) |  |  |  |
| Yes | 2.30 | 1.43 | 0.107 |
| Practice location (Ref. province not adjacent to the capital) |  |  |  |
| Province adjacent to the capital | -4.00 | 1.35 | 0.003 |
| Residence (Ref. Rural) |  |  |  |
| Urban | 3.24 | 1.88 | 0.085 |
| Practice site (Ref. Rural) |  |  |  |
| Urban | -5.04 | 1.90 | 0.008 |
| Gender (Ref. Female) |  |  |  |
| Male | -1.70 | 1.91 | 0.375 |
| Length of clinical experience (Ref. < 20 yrs) |  |  |  |
| > 20 yrs | -0.69 | 2.16 | 0.748 |
| Residency program completed (Ref. family medicine) |  |  |  |
| Internal medicine | -3.61 | 1.59 | 0.024 |
| Other | -5.04 | 2.20 | 0.022 |

**Supplementary Table 2: Association between provider characteristics and attitude score from a linear regression**

| Variable | Coef. | S.E. | p-value |
| --- | --- | --- | --- |
| Intercept | 62.02 | 4.71 | <0.001 |
| Age (Ref. 25-34 years) |  |  |  |
| 35-44 years | -1.16 | 5.02 | 0.818 |
| 45-54 years | -4.95 | 4.86 | 0.309 |
| 55-64 years | 0.41 | 5.46 | 0.941 |
| 65+ years | -2.92 | 5.79 | 0.615 |
| Participation in the USAID retraining program (Ref. No.) |  |  |  |
| Yes | -5.15 | 2.22 | 0.021 |
| Practice location (Ref. province not adjacent to the capital) |  |  |  |
| Province adjacent to the capital | -1.02 | 2.10 | 0.627 |
| Residence (Ref. Rural) |  |  |  |
| Urban | 1.06 | 2.92 | 0.716 |
| Practice site (Ref. Rural) |  |  |  |
| Urban | -2.87 | 2.95 | 0.331 |
| Gender (Ref. Female) |  |  |  |
| Male | -3.05 | 2.98 | 0.307 |
| Length of clinical experience (Ref. < 20 yrs) |  |  |  |
| > 20 yrs | 4.99 | 3.36 | 0.138 |
| Residency program completed (Ref. family medicine) |  |  |  |
| Internal medicine | -13.1 | 2.48 | <0.001 |
| Other | -12.1 | 3.42 | <0.001 |

**Supplementary Table 3: Association between provider characteristics and practice score from a linear regression**

| Variable | Coef. | S.E. | p-value |
| --- | --- | --- | --- |
| Intercept | 76.82 | 1.46 | <0.001 |
| Age (Ref. 25-34 years) |  |  |  |
| 35-44 years | -0.66 | 1.56 | 0.109 |
| 45-54 years | -2.51 | 1.51 | 0.110 |
| 55-64 years | -2.42 | 1.70 | 0.816 |
| 65+ years | -0.40 | 1.80 | 0.592 |
| Participation in the USAID retraining program (Ref. No.) |  |  |  |
| Yes | 0.21 | 0.69 | 0.764 |
| Practice location (Ref. province not adjacent to the capital) |  |  |  |
| Province adjacent to the capital | -0.66 | 0.65 | 0.315 |
| Residence (Ref. Rural) |  |  |  |
| Urban | 1.11 | 0.91 | 0.223 |
| Practice site (Ref. Rural) |  |  |  |
| Urban | 1.55 | 0.92 | 0.091 |
| Gender (Ref. Female) |  |  |  |
| Male | -0.72 | 0.92 | 0.434 |
| Length of clinical experience (Ref. < 20 yrs) |  |  |  |
| > 20 yrs | -0.41 | 1.04 | 0.696 |
| Residency program completed (Ref. family medicine) |  |  |  |
| Internal medicine | -3.26 | 0.77 | <0.001 |
| Other | -1.44 | 1.06 | 0.176 |

**Supplementary Table 4: Association between provider characteristics and priority score from a linear regression**

| Variable | Coef. | S.E. | p-value |
| --- | --- | --- | --- |
| Intercept | 93.83 | 2.11 | <0.001 |
| Age (Ref. 25-34 years) |  |  |  |
| 35-44 years | -1.73 | 2.25 | 0.443 |
| 45-54 years | -0.82 | 2.18 | 0.708 |
| 55-64 years | -0.03 | 2.45 | 0.990 |
| 65+ years | 1.28 | 2.59 | 0.622 |
| Participation in the USAID retraining program (Ref. No) |  |  |  |
| Yes | -0.56 | 1.00 | 0.574 |
| Practice location (Ref. province not adjacent to the capital) |  |  |  |
| Province adjacent to the capital | 1.68 | 0.94 | 0.075 |
| Residence (Ref. Rural) |  |  |  |
| Urban | 1.46 | 1.31 | 0.266 |
| Practice site (Ref. Rural) |  |  |  |
| Urban | -1.30 | 1.32 | 0.326 |
| Gender (Ref. Female) |  |  |  |
| Male | 0.90 | 1.33 | 0.501 |
| Length of clinical experience (Ref. < 20 yrs) |  |  |  |
| > 20 yrs | -0.89 | 1.51 | 0.556 |
| Residency program completed (Ref. family medicine) |  |  |  |
| Internal medicine | -2.88 | 1.11 | 0.010 |
| Other | -0.12 | 1.53 | 0.937 |

**Supplementary Table 5: Association between provider characteristics and confidence score from a linear regression**

| Variable | Coef. | S.E. | p-value |
| --- | --- | --- | --- |
| Intercept | 70.83 | 9.10 | <0.001 |
| Age (Ref. 25-34 years) |  |  |  |
| 35-44 years | -10.86 | 9.71 | 0.264 |
| 45-54 years | -13.73 | 9.41 | 0.145 |
| 55-64 years | -12.44 | 10.57 | 0.240 |
| 65+ years | -14.00 | 11.20 | 0.212 |
| Participation in the USAID retraining program (Ref. No.) |  |  |  |
| Yes | -15.31 | 4.30 | <0.001 |
| Practice location (Ref. province not adjacent to the capital) |  |  |  |
| Province adjacent to the capital | 22.76 | 4.07 | <0.001 |
| Residence (Ref. Rural) |  |  |  |
| Urban | -1.73 | 5.65 | 0.760 |
| Practice site (Ref. Rural) |  |  |  |
| Urban | -0.54 | 5.71 | 0.925 |
| Gender (Ref. Female) |  |  |  |
| Male | -8.10 | 5.76 | 0.161 |
| Length of clinical experience (Ref. < 20 yrs) |  |  |  |
| > 20 yrs | 9.71 | 6.50 | 0.136 |
| Residency program completed (Ref. family medicine) |  |  |  |
| Internal medicine | -14.39 | 4.79 | 0.003 |
| Other | -15.56 | 6.61 | 0.019 |
