# Supplementary material for: Assessment of knowledge, attitudes, and practices of primary healthcare physicians in provinces of Armenia towards hypertension management: a cross-sectional study
Source: BMC Prim Care. 2026 May 8;27:230. doi: 10.1186/s12875-026-03359-6 (PMC13262312; doi:10.1186/s12875-026-03359-6)
Supplement: Supplementary file 2 — Supplementary Material 2. [file 12875_2026_3359_MOESM2_ESM.docx]

**Checklist**

| **Section / Topic** | **No** | **Checklist item description** | **Reported on page no.** |
| --- | --- | --- | --- |
| **Title and abstract** | | |  |
| Title and structured abstract | 1 | Structured summary of the trial design, methods, results, and conclusions. | Pages 1-3 |
| **Introduction** | | |  |
| Background and rationale | 2 | Study background and rationale. | Pages 4-5 |
| **Methods** | | |  |
| Study Population | 3 | Details of conduct and reporting of the trial. Eligibility criteria for participants. How sample size was determined, including all assumptions supporting the sample size calculation. Settings (e.g., community, hospital) and locations (e.g., countries, sites) where the trial was conducted. | Page 6 |
| Survey design | 4 | Details of survey methods and administration. | Page 7 |
| Statistical analysis | 5 | Statistical methods used to assess the association between demographic characteristics. | Page 8 |
| **Results** | | |  |
| Knowledge | 6 | Structured details of knowledge-related responses and findings. | Pages 9-16 |
| Attitude | 7 | Structured details of attitude-related responses and findings. | Pages 17-19 |
| Practice | 8 | Structured details of practice-related responses and findings. | Pages 19-23 |
| Priority | 9 | Structured details of priority-related responses and findings. | Pages 23-26 |
| Confidence | 10 | Structured details of confidence-related responses and findings. | Pages 27-29 |
| Provider Characteristics, Consultation times, Referral patterns | 11 | Strength of associations between provider characteristics and KAP-related domains. Impact of USAID training on consultation times and referral patterns. | Pages 29-35 |
| **Discussion** | | |  |
| Interpretation and Analysis | 12 | Interpretation and analysis of study findings, consistent with results. | Pages 36-39 |
| Conclusion | 13 | Conclusion and highlights of study findings. | Page 39 |
